# Quantitative assessment of human health risks from chemical pollution in the uMsunduzi River, South Africa

**DOI:** 10.1007/s11356-023-30534-4

**Published:** 2023-10-24

**Authors:** Zesizwe Ngubane, Bloodless Dzwairo, Brenda Moodley, Thor Axel Stenström, Ekaterina Sokolova

**Affiliations:** 1 Department of Civil Engineering, Midlands, Durban University of Technology, Pietermaritzburg, South Africa; 2 Institute for Water and Wastewater Technology, Durban University of Technology, Durban, South Africa; 3 School of Chemistry and Physics, University of KwaZulu-Natal, Westville Campus, Durban, South Africa; 4 Department of Earth Sciences, Uppsala University, Uppsala, Sweden

**Keywords:** Heavy metals, Nitrates, Organochlorinated pesticides, Pharmaceuticals and personal care products, Phosphates, Quantitative chemical risk assessment

## Abstract

A quantitative chemical risk assessment was performed using published data as well as data from the official monitoring programme for the uMsunduzi River in KwaZulu-Natal, South Africa. The chemicals assessed were organochlorinated pesticides (OCPs), pharmaceuticals and personal care products (PPCPs), heavy metals, and nitrates and phosphates. The water from uMsunduzi River is used locally without treatment. Consequently, the exposure routes investigated were via ingestion during domestic drinking and incidental ingestion during recreational activities, which were swimming and non-competitive canoeing, for both adults and children. For the individual chemicals, non-carcinogenic risks using the hazard quotient (HQ) and carcinogenic risks using the cancer risk (CR) were quantified. It was found that the exposed population is likely to experience non-carcinogenic effects from pesticides and phosphates, but not from PPCPs, heavy metals and nitrates. This study also found that the carcinogenic risks for OCPs were higher than the tolerable limit of 10^-5^, while for lead the risk was below the tolerable limit. Some of the activities that potentially contribute to chemicals onto the uMsunduzi River are subsistence farming, small plantations, illegal dumping, industries, and broken sewers. The findings of this study may act as the technical foundation for the introduction of pollution reduction measures within the catchment, including public education.

**Graphical Abstract:**

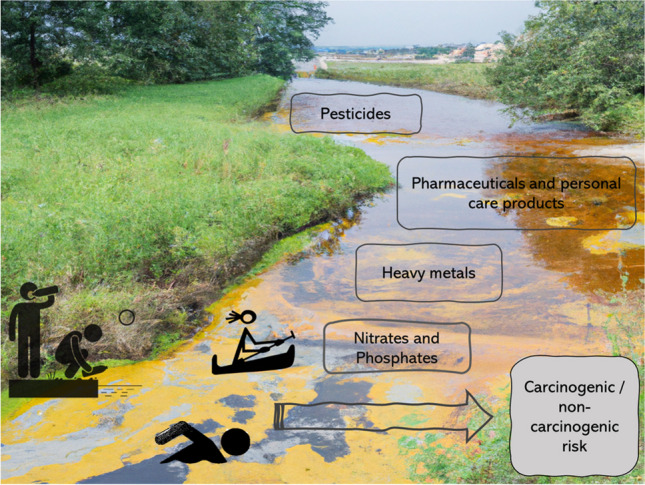

**Supplementary Information:**

The online version contains supplementary material available at 10.1007/s11356-023-30534-4.

## Introduction

According to the World Health Organisation (WHO 2020), 24% of deaths worldwide are caused by environmental factors that can be modified, including exposure to toxic chemicals. Chemicals have an impact on aquatic environments in a variety of ways, including through wastewater and industrial discharges, agricultural residues, and land runoff. The conventional treatment processes offered by water or wastewater treatment plants cannot generally remove or reduce these substances to acceptable low values. Ecological studies, animal models, human clinical observations, and epidemiological studies confirm the importance of chemicals impacting wildlife and humans (Pironti et al. 2021). Several studies have studied the occurrence of these chemicals in wastewater (for example, Adeyinka et al. 2019; Nyamukamba et al. 2019), surface water (for example, Matongo et al. 2015; Sengar and Vijayanandan 2022), sediments (for example Shozi 2015; Adeyinka et al. 2019), and groundwater (Zhai et al. 2017; Mohammadi et al. 2019).

In the surface waters of the uMsunduzi River, South Africa, organochlorinated pesticides (OCPs), pharmaceuticals and personal care products (PPCPs), heavy metals, and nitrates and phosphates have been detected in variable concentrations (Matongo et al. 2015; Shozi 2015; Adeyinka et al. 2019). In addition, chemical spills have been observed in the uMsunduzi River and its tributaries. To illustrate, in the year 2019, an oil spill from an oil company was reported; the spill affected the majority of the uMsunduzi River downstream of the city of Pietermaritzburg (Mdletshe 2019). Fish, aquatic animals, and livestock who drank from the river died as a result of this spill (Mdletshe 2019). Additionally, the different settlements within the catchment use this river for recreational activities and drinking without treatment.

In their assessment of forty research studies that investigated agrochemicals in freshwater aquatic habitats in South Africa from 2011 to 2020, Horak et al. (2021) examined rivers such as the uMngeni, Vaal, Olifants, Buffalo, Lourens, and uMsunduzi. According to these research studies, agrochemicals were found in all of South Africa's provinces, including along the Indian Ocean coast. OCPs are a group of chlorinated chemicals historically used as insecticides with some classified as persistent organic pollutants based on their extended half-lives in the environment (Adeyinka et al. 2019; Wolmarans et al. 2021) and toxicity even at low concentrations (de Souza et al. 2020). These pesticides were banned or severely restricted by the Stockholm Convention on Persistent Organic Pollutants in the year 1983 (UNEP 2020). Agrochemicals can impair organisms' (including humans) ability to produce hormones normally, which can result in several endocrine disrupting impacts (Qu et al. 2015), including intersex, decreased spermatogenesis, asymmetric urogenital papillae, testicular lesions, and infertile eggs (Horak et al. 2021). As a result, OCPs continue to pose a hazard to both the ecological environment and human health (Chen et al. 2020). In humans, OCPs have also been listed as supposed carcinogens (Wexler 2014).

According to Adeleye et al. (2022), PPCPs are introduced into urban wastewater systems through hospitals, PPCPs producers, and agricultural sources in addition to human excretion and typical household usage. Based on the data obtained from wastewater treatment plants (WWTPs) in South Africa, analgesics, antibiotics, and stimulants are the most abundant PPCPs in raw wastewater (Adeleye et al. 2022). Regulation of pharmaceuticals and their treatment are not as stringent in African countries as compared to developed economies; and the current wastewater treatment systems were not designed with the intent of managing pharmaceuticals as pollutants (Agunbiade and Moodley 2016) but will, to a varying degree, reduce their concentrations (Matongo et al. 2015; Faleye et al. 2019). Different investigations confirm the presence of PPCPs in natural waters around the world, including freshwater (such as rivers, streams, lakes), marine and estuarine environments, groundwater, and sediment (Agunbiade and Moodley 2016; Kong et al. 2021; Adeleye et al. 2022). The presence of PPCPs in receiving waters and sediments has been shown in the KwaZulu-Natal province, South Africa (Matongo et al. 2015; Agunbiade and Moodley 2016). In the environment, PPCPs have been reported to be enriched highly in organisms and amplified with the food chain (Lin et al. 2023). In humans, PPCPs have different side effects upon prolonged exposure and overdosage (Wexler 2014).

Heavy metal ions are among the most released pollutants, and for this reason as well as their persistence they are particularly concerning. About 40% of lakes and rivers on Earth are polluted with heavy metals, the sources of which are both natural and anthropogenic (Zamora-Ledezma et al. 2021). Particularly in recent decades, human activities such as urbanisation, industrialisation, and pollution have increased the concentration of these pollutants (Zamora-Ledezma et al. 2021). Mean concentrations of heavy metals in water samples from South Africa exceeded the World Health Organization guidelines for safe levels of intake, according to the study performed by Genthe et al. (2018). That study followed the death of a dozen crocodiles in the Olifants River catchment near the South Africa-Mozambique border, where it was found that the death was due to anthropogenic pollution (Genthe et al. 2018). In humans, these chemicals have such effects as liver damage, reduced lung function, and thyroid disorder with some heavy metals having probable carcinogenic effects (Wexler 2014).

Agricultural runoff, industrial effluents, and municipal wastewater systems may lead to excess nitrogen and phosphorus load in water environments. As a result, algal overgrowths or the presence of noxious algal species can become a nuisance and interfere with the desirable uses of a water body (DWAF 1996b). Waterbody eutrophication in turn impacts human health by causing conjunctivitis, dermatological conditions, and gastrointestinal illnesses (Oberholster and Ashton 2008).

The human health risk assessment is the process used to estimate the nature and probability of adverse health effects among people exposed to hazardous environmental substances now or in the future (Genthe et al. 2018). To understand the nature, magnitude, and probability of an adverse health or environmental effect of a chemical, a chemical risk assessment is required (Nyamukamba et al. 2019; Moloi et al. 2020). Health risk assessments are primarily intended to protect consumers against serious adverse effects of toxicants in food or water (Taiwo 2019) and comprise four main stages: hazard identification, exposure assessment, dose-response characterisation, and risk characterisation (Fryer et al. 2006). Chen et al. (2020) performed an ecological and health risk assessment of OCPs in an urbanised river network of Shanghai, China. Sengar and Vijayanandan (2022) assessed ecological and human health risks of PPCPs detected in surface waters and wastewater in India. Carcinogenic and non-carcinogenic health risks posed by heavy metals were assessed in groundwater water in rural Iran (Maleki and Jari 2021), in wastewater discharge in the Vaal River Basin in South Africa (Moloi et al. 2020), and in drinking water in Khorramabad, Iran (Mohammadi et al. 2019). The health impact of nitrate pollution in groundwater was investigated by Zhai et al. (2017) in Songnen Plain of Northeast China, and in this study it was concluded that risk levels generally followed the pattern of being highest for infants, followed by children, adult females, and then adult males.

The goal of this study is to demonstrate the importance of riverine chemical pollution by undertaking a quantitative chemical risk assessment for consumers of untreated water engaged in domestic and recreational activities. This strategy helps identify important areas of concern while also highlighting the need for mitigation measures. The present study aims, therefore, to quantify the carcinogenic and non-carcinogenic risks that chemical pollutants pose to the population of the uMsunduzi catchment through consumption of the water for drinking (without treatment) and inadvertent ingestion during swimming and canoeing. The specific objectives are to (i) peruse existing literature on chemical concentrations within the uMsunduzi River, (ii) access and evaluate river monitoring data, and (iii) quantify the probability of developing both adverse non-carcinogenic health effects (hazard quotient: HQ) and cancer risk (CR).

## Methods and Materials

### Study area

**Fig. 1:**
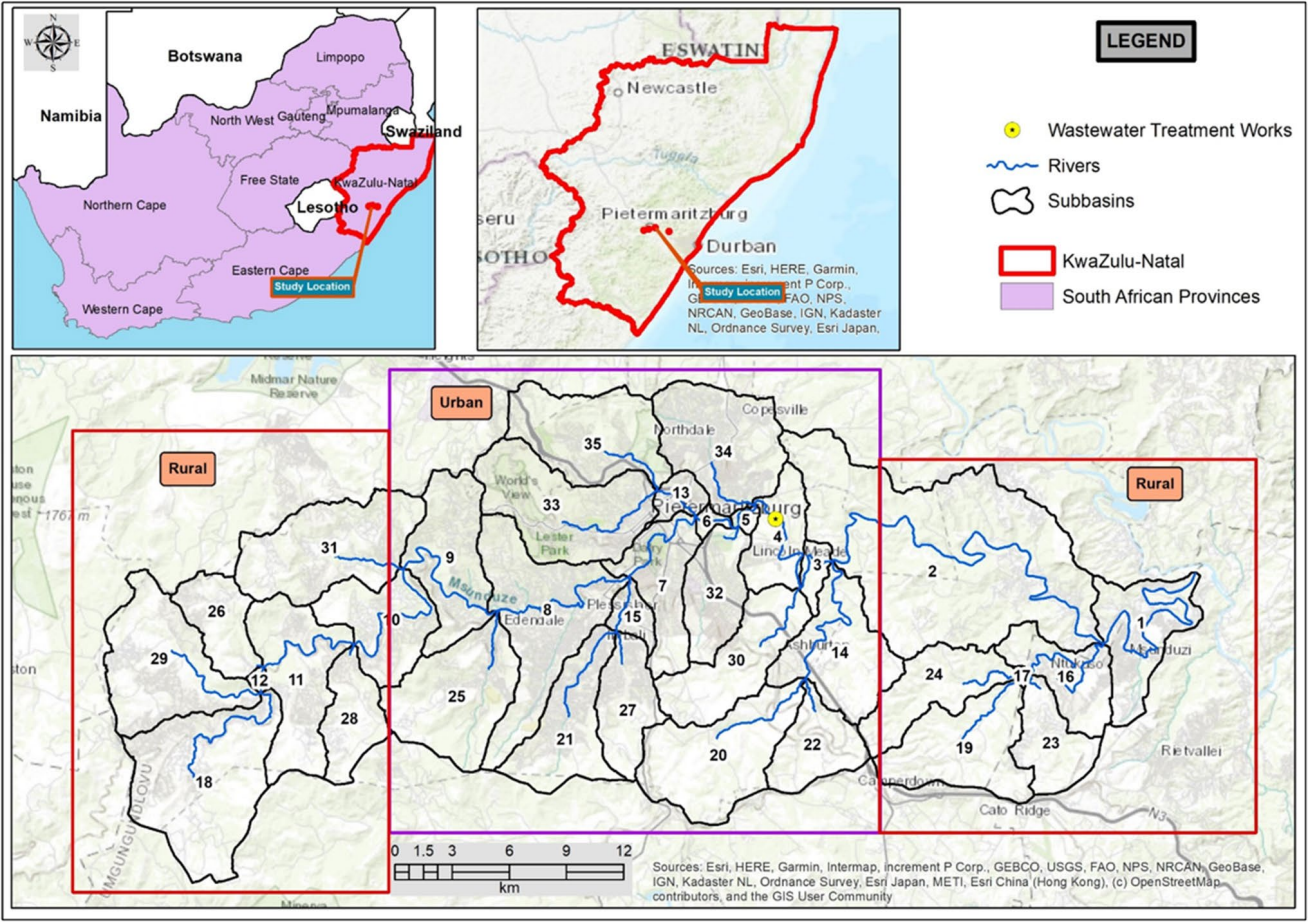
UMsunduzi catchment location. Please follow the link to view in full layout: https://www.google.com/maps/d/viewer?mid=1NQXdFcqDjShMQxRgn4p8ILzpj42NpgdG&ll=-29.63672034343441%2C30.475072992298692&z=11

The 875 km^2^ uMsunduzi catchment is in the KwaZulu-Natal province, South Africa. The uMsunduzi River has a 115 km watercourse length and passes through the city of Pietermaritzburg (29°37’S 30°23’E) and is a major tributary of the uMngeni River. Pietermaritzburg is the second largest city in the province of KwaZulu-Natal. UMsunduzi Municipality has an estimated population of 600 000. UMsunduzi River flows through rural and urban dwellings as seen in Fig. 1.

### Chemicals detected within the uMsunduzi catchment

#### Organochlorinated Pesticides

Adeyinka et al. (2019) evaluated the concentrations of OCPs in the sediments, soil, and surface water of the uMsunduzi catchment, and at the Darvill wastewater treatment plant. The pesticides detected in the uMsunduzi River were: hexachlorobenzene (HCB), hexachlorocyclohexane (HCH), heptachlor, aldrin, dichlorodiphenyldichloroethane (o,p’-DDD, p,p’-DDD), dichlorodiphenyltrichloroethane (o,p’-DDT, p,p’-DDT), dieldrin, endrin, and mirex (Adeyinka et al. 2019). Two samples (per location) were collected, and the concentrations are presented in Table 1 as reported by the authors. A grab sampling technique was used to collect wastewater or surface water samples from a depth of 1–2 cm from the water surface.

**Table 1:** Concentrations of organochlorinated pesticides presented as mean (*SD*) (mg/L) detected in selected sub-basins of the uMsunduzi River surface water by Adeyinka et al. (2019). (NGV = No guideline value)

Pesticide	Subbasin 10	Subbasin 8	Subbasin 4	Subbasin 1	DWAF Guidelines (mg/L) (DWAF 1996a; Horak et al. 2021)	WHO Guidelines (mg/L) (WHO 2017)
HCB	3.31 (0.86)	0.76 (0.02)	1.73 (0.49)	1.13 (1.99)	NGV	NGV
HCH	8.77 (1.15)	11.30 (0.50)	17.63 (0.66)	6.67 (2.75)	0.015	0.002
Heptachlor	2.55 (1.14)	6.42 (0.31)	0.06 (1.86)	3.44 (0.12)	<0.39	NGV
Aldrin	3.72 (0.80)	7.71 (0.27)	1.89 (2.16)	4.64 (0.09)	0.01	3.00x10^-5^
o,p’-DDE	9.17 (0.30)	6.73 (0.32)	7.48 (0.47)	7.48 (0.62)	0.0015	0.001
p.p’-DDE	14.36 (0.29)	12.6 (0.44)	15.06 (0.51)	11.81 (0.10)	0.0015	0.001
o.p’-DDD	16.36 (0.23)	14.24 (0.05)	15.14 (0.43)	14.55 (0.12)	0.0015	0.001
Dieldrin	8.39 (0.19)	6.54 (0.28)	8.14 (0.64)	6.84 (0.18)	0.0015	0.001
Endrin	2.95 (1.32)	10.15 (0.26)	0.37 (1.31)	2.04 (0.83)	0.005	3.00x10^-5^
p,p’-DDD	6.71 (1.21)	12.35 (0.96)	2.93 (1.55)	4.82 (1.13)	0.0015	0.001
o,p’- DDT	1.94 (2.16)	7.76 (1.50)	0.98 (0.24)	1.52 (1.50)	0.0015	0.001
p,p’-DDT	13.24 (0.23)	13.53 (0.31)	2.09 (0.52)	18.84 (0.50)	0.0015	0.001
Mirex	16.79 (0.27)	24.98 (0.41)	12.08 (1.33)	19.06 (0.13)	0.001	NGV

As supplementary information, Table S1 shows the respective OCP effects on humans and the status of OCPs in South Africa. Table 1 shows the drinking water guidelines set by the South African Department of Water Affairs Guidelines (DWAF 1996a) as well as those set by the World Health Organisation (WHO 2017) against these pesticides. The Encyclopaedia of Toxicology (Wexler 2014) summarises the carcinogenic effects of different pesticides, and only endrin is listed as not carcinogenic. While most OCPs have been banned worldwide, they are still being used illegally for agricultural use in some countries, including South Africa. Their presence may also be attributed to their long half-lives in the environment as indicated in supplementary Table S1.

#### Pharmaceuticals and Personal Care Products

The PPCPs in the uMsunduzi catchment were measured by Agunbiade and Moodley (2016) and Matongo et al. (2015), their concentrations are shown in Table 2. These include therapeutic classes such as antipyretics (acetaminophen, aspirin, diclofenac, ibuprofen, ketoprofen); stimulants (caffeine); anti-epileptics (carbamazepine); psychotics (clozapine); antibiotics (ampicillin, ciprofloxacin, erythromycin, metronidazole, nalidixic acid, sulfamethoxazole, sulfamethazine, trimethoprim); and antihyperlipidemic (bezafibrate), as shown in Table S2. These were selected based on statistics of drug usage in South Africa (Agunbiade and Moodley 2016). Sampling was performed over spring, summer, autumn, and winter with one sample collected per location for each season in one year. A grab sampling technique was used to collect wastewater or surface water samples from a depth of 1–2 cm from the water surface. The presented concentrations show the mean of the four samples and the standard deviation for the work of Matongo et al. (2015) as reported by the authors. For Agunbiade and Moodley (2016), the mean concentrations are shown, as their work only reported mean concentrations for surface water. Where both Matongo et al. (2015) and Agunbiade and Moodley (2016) have the same PPCP, for instance, ibuprofen, the higher of the two values was used to calculate risk.

**Table 2:** Concentrations of pharmaceuticals and personal care products presented as mean (*SD*) (μg/L) detected in selected sub-basins of the uMsunduzi River surface water by Matongo et al. (2015)^A^ and Agunbiade and Moodley (2016)^B^ (ND = Not Detected)

Therapeutic Class	Pharmaceutical	Subbasin 10	Subbasin 8	Subbasin 4	Subbasin 1	Reference
Antipyretics	Acetaminophen	0.99 (5.35)	1.29 (0.57)	1.26 (3.47)	1.74 (4.35)	A
	Aspirin	13.70	14.54	13.84	25.35	B
	Diclofenac	0.88	8.17	0.60	2.08	B
	Ibuprofen	0.55	0.66	0.70	0.45	B
		84.6 (6.65)	27.6 (0.63)	4.7 (1.43)	2.58 (0.76)	A
	Ketoprofen	0.39	ND	0.45	ND	B
Stimulants	Caffeine	0.11 (3.45)	ND	ND	3.32 (0.98)	A
Anti-epileptics	Carbamazepine	1.26 (7.65)	3.24 (0.67)	0.29 (3.95)	0.32 (2.54)	B
Psychotics	Clozapine	8.89 (4.56)	5.59 (0.33)	2.18 (0.57)	2.48 (7.65)	A
Antibiotics	Ampicillin	3.68	4.05	3.87	3.21	B
	Ciprofloxacin	2.63	12.99	14.33	2.40	B
	Erythromycin	0.06 (13.56)	ND	ND	ND	A
	Nalidixic acid	19.42	12.48	14.90	20.66	B
	Sulfamethoxazole	ND	1.22 (3.75)	4.32 (0.56)	ND	A
	Sulfamethazine	ND	ND	ND	1.09	A
	Trimethoprim	0.29 (0.48)	ND	ND	ND	A
Antihyperlipidemic	Bezafibrate	0.23	ND	0.31	ND	B

The recommended reference dose (RfD) shown in the supplementary Table S2 derived from the National Department of Health of South Africa (2020) shows that seven of the drugs are not recommended for children.

#### Heavy Metals and Nutrients

The heavy metals chosen for risk analysis were primarily determined by the studies referenced, but it is advisable to consider additional heavy metals when assessing risks. The concentrations of heavy metals and nutrients were measured in selected subbasins of the uMsunduzi River surface water by Shozi (2015) and Umgeni Water respectively (Table 3). The metals detected by Shozi (2015) were copper, lead, and zinc during a once-off sampling event in September 2013, when one sample was collected per chosen site; these data were presented as a single value in Table 3 below. A grab sampling technique was used to collect wastewater or surface water samples from a depth of 1–2 cm from the water surface. As part of Umgeni Water monitoring programme, concentrations of nitrates and soluble reactive phosphates have been quantified in the uMsunduzi River at different sites. The mean concentrations and their standard deviations for nitrates and phosphates (Table 3) were calculated over the period between January 1990 and December 2018; sampling frequency varied, with number of samples ranging from 149 to 1490 between sampling stations and chemicals. Some possible human effects due to exposure to these heavy metals and nutrients are listed in the supplementary Table S3 after Wexler (2014).

**Table 3:** Concentrations of heavy metals and nutrients detected in selected subbasins of the uMsunduzi River surface water by Shozi (2015) for heavy metals and Umgeni Water for nutrients presented as mean (*SD*) (mg/L**)**. (NGV = No Guideline Value and ND = Not Detected)

Chemical	Subbasin 10	Subbasin 8	Subbasin 4	Subbasin 1	DWAF Guidelines (mg/L) (DWAF 1996a)	WHO Guidelines (mg/L) (WHO 2017)
Copper	5.92 x 10^-5^	1.87 x 10^-5^	ND	-	30	2
Lead	4.53 x 10^-3^	1.47 x 10^-4^	5.65 x 10^-5^	-	0.01	0.01
Zinc	8.10 x 10^-4^	3.13 x 10^-4^	ND	-	10	NGV
Nitrates	0.93 (0.41)	1.06 (0.49)	1.85 (2.18)	2.37 (1.30)	10	10
Phosphates	0.007 (0.013)	0.02 (0.04)	0.21 (0.30)	0.18 (0.21)	NGV	NGV

### Risk Assessment

Studies, locally and abroad, that have quantified chemical risk via ingestion have looked at treated water, while this study assumed no treatment of the water, as this is the normality in the studied communities. The chemical concentrations in the surface waters of the uMsunduzi River used in the risk assessment calculations were based on the following sources: organochlorinated pesticides (Adeyinka et al. 2019), pharmaceuticals and personal care products (Matongo et al. 2015; Agunbiade and Moodley 2016), heavy metals (Shozi 2015), and nitrates and phosphates (Umgeni Water monitoring data). Ngubane et al. (2022), our earlier study in which we estimated microbial hazards in this watershed, provided the exposed population and the exposure routes employed in this research. However, for the sake of simplicity, only maximum ingestion rates (IR) were used in the current analysis. The exposure routes investigated were direct ingestion of the uMsunduzi River water during recreational swimming, canoeing training, and drinking.

Recreational swimming was considered for subbasins 1, 4, 8, and 10. This is because it was observed during the study that the population along this stretch of the catchment habitually swim in the river during warm periods. The exposed population was categorised into children and adults. The volume ingested during swimming was estimated based on the ranges of 37-47 mL for children and 16-24 mL for adults per 45 minute event as reported by Dufour et al. (2006). The values used in the current study were 0.0627 (children) and 0.032 (adults) L/day. Swimming was assumed to take place 50 times a year of an hour’s swim, during warm periods.

Canoeing was considered for subbasin 8 as training takes place in this stretch of the river. Training information was obtained from Mr. Z. L. Mthalane (*pers. comm.*, 2019), a coach at two canoe clubs and a seasoned Dusi Canoe Marathon and Dusi Non-stop participant. Approximately 1000-2000 paddlers enter the Dusi Canoe Marathon annually. The exposed population was categorised into two: children (10 - 18 years old) and adults (>18 years old) based on the competition categories and the required hours for training. Based on the training schedules, 0.5 to 1.5 hours (children), and 1.5 to 2 hours (adults) per day were assumed. Forty days of canoeing were assumed per year. The ingestion volume of 5.8 mL per 45 minute event (Dorevitch et al. 2011) was used as a baseline to estimate the ingested volumes during canoeing events. The values used in this study were 0.0116 (children) and 0.0154 (adults) L/day.

Drinking water was considered for subbasins 10 and 1, which are in the upper and lower rural parts of the catchment, respectively. The exposed population was categorised into children and adults. The daily ingestion volumes for South Africans were adapted from Steyn et al. (2001), and the resulting values used were: 0.773 (children) and 0.952 (adults) L/day.

The risk to develop adverse health effects due to exposure to chemical substances is estimated using hazard quotient (HQ) for harmful non-carcinogenic effects. HQ is the ratio of Chronic Daily Intake (CDI) to Reference Dose (RfD) as calculated using Equation 1 (Pieters and Horn 2020). A value of HQ below 1 means that the exposed population is unlikely to experience adverse health effects, and an HQ value greater than 1 represents a potential health risk to the exposed population (USEPA 2009). CDI is the potential exposure to a substance, and RfD is the level at which no adverse effects are expected. CDI was calculated using Equation 2.

1 $$HQ=\frac{CDI}{RfD}$$

Where CDI is the Chronic Daily Intake (mg/kg/day) via ingestion, and RfD is the recommended dose.

2 $$CDI=\frac{Cw\ \times\ IR\ \times\ EF\ \times\ ED}{BM\ \times\ AT}$$

Where Cw is the concentration of the chemical (mg/L) in ingested water, IR is the ingestion rate (L/day), EF is the exposure frequency (day/year), ED is the exposure duration (years), BM is the human body mass (kg), AT is the average time (days).

In this study, RfD values for OCPs were based on the Agency for Toxic Substances and Disease Registry (ATSDR 2005, 2007, 2015, 2020); for PPCPs they were based on the South African National Department of Health guidelines as published in the National Department of Health of South Africa (2020); for heavy metals, nitrates and phosphates, RfD values were based on the South African National Standards (SANS 2015). The body mass (BM) of 66 kg for adults and 35 kg for children was based on the local study by Pieters and Horn (2020); with the corresponding 70 years and 12 years exposure duration (ED). The Exposure frequency (EF) was set at 50, 365, and 40 days per year for swimming, drinking, and canoeing, respectively.

According to WHO (2010), incremental lifetime cancer risk (ILCR) refers to the incremental risk a person faces over a lifetime because of exposure to a given concentration of a carcinogenic agent averaged over a lifetime. ILCR is estimated using Equation 3 after WHO (2010). The World Health Organization (WHO 2008) and several countries worldwide have set their acceptable cancer risk level at 10^-5^ for 70 years life expectancy.

3 $$ILCR= CDI\times CSF$$

Where, CSF (mg/kg/day) is the cancer slope factor and is defined as the risk generated by a lifetime average amount of one mg/kg/day of carcinogenic chemical and is pollutant specific (Qu et al. 2015). In this study, CSF values for chemicals with potential carcinogenic effects were based on various toxicological data as per supplementary Tables S1 and S3.

## Results

**Fig. 2:**
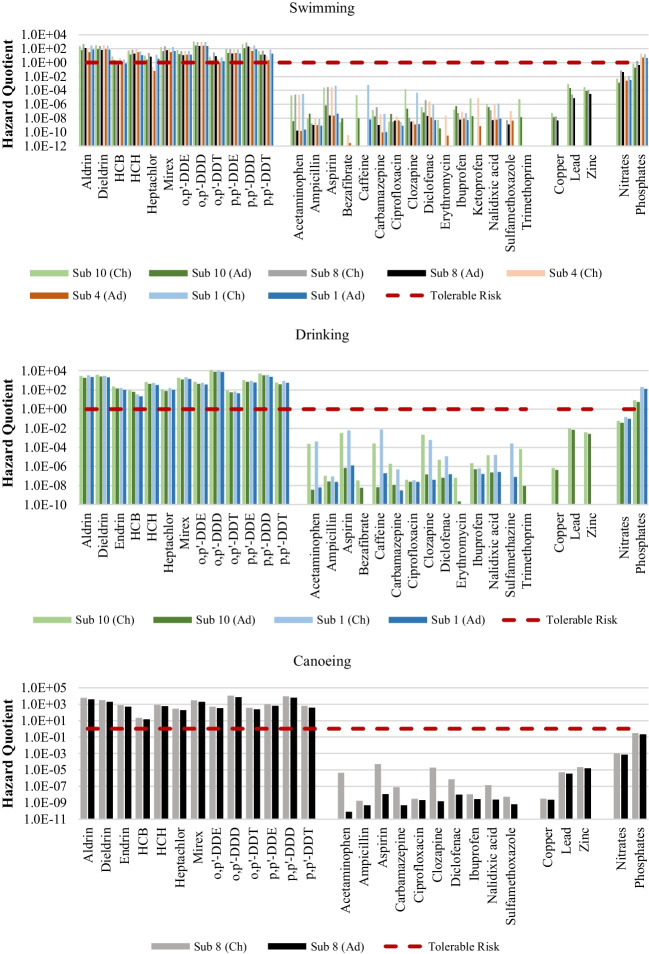
Hazard quotient calculated using the mean concentrations for non-carcinogenic risk during swimming, drinking, and canoeing (top to bottom, respectively) for both children (Ch) and adults (Ad)

Figure 2 shows HQ values representing non-carcinogenic risks of all chemicals calculated using the mean concentrations considering swimming, drinking, and canoeing as exposure routes. Since PPCPs, heavy metals, and nitrates had HQ values below 1, the exposed population is unlikely to experience adverse non-carcinogenic health effects due to ingestion of these chemicals. In contrast, for pesticides, HQ values exceeded 1 for all exposure routes, suggesting possible non-carcinogenic health risks, which may lead to increased risk of hospitalisations and death (Wexler 2014). The health risks associated with consuming water containing high phosphate levels are typically influenced by the overall water quality and the presence of other harmful substances, rather than being solely attributed to phosphates. For instance, algal blooms are caused by the simultaneous presence of N and P (Chen et al. 2020). Algal overgrowths or the presence of noxious algal species can, however, become a nuisance and interfere with the desirable uses of a water body (DWAF 1996b). Eutrophication of waterbodies has a negative effect on human health, causing gastrointestinal diseases, dermatological disorders, and conjunctivitis (DWAF 1996b). For PPCPs, OCPs, and nutrients an upper bound estimate for HQ was also calculated using mean measured concentration plus standard deviation (Figure S1 in the supplementary material), but the same could not be performed for heavy metals due to the lack of data. The HQ increased, overall, with HQ for carbamazepine exceeding 1. This means that, if the variability in the concentrations is considered, as opposed to when only mean values are considered, PPCPs can pose non-carcinogenic health effects to the exposed population.

**Fig. 3:**
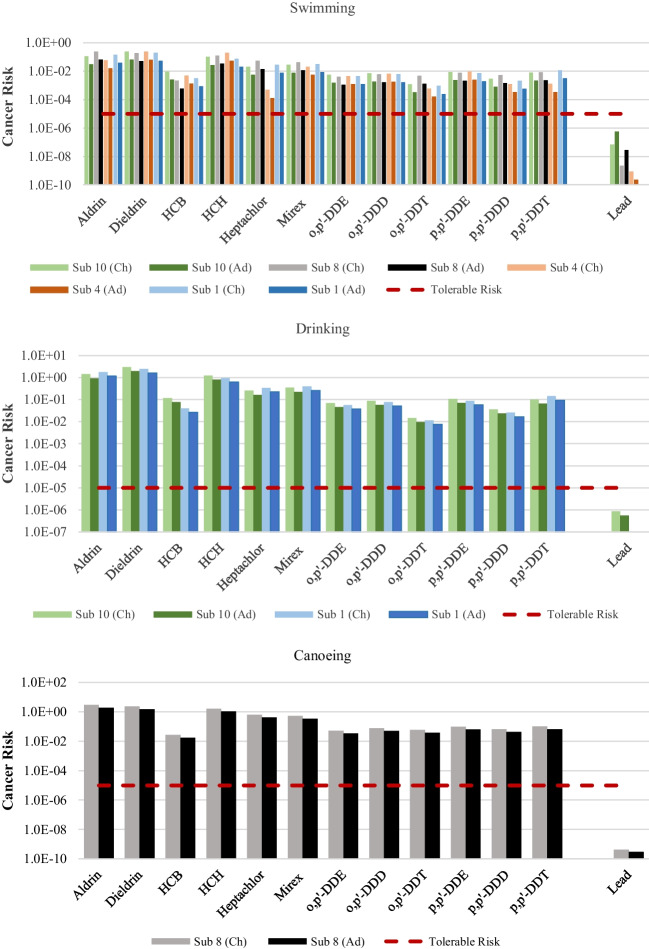
Cancer risk calculated using mean concentrations for carcinogenic risk during swimming, drinking, and canoeing (top to bottom, respectively) for both children (Ch) and adults (Ad)

The results for cancer risk, using the mean concentrations, are shown in Fig. 3. Apart from endrin, all pesticides have the probability to cause cancer to the exposed population in all subbasins and exposure routes, with cancer risk greater than 10^-5^. Dieldrin and aldrin pose the highest risk in all exposure scenarios in subbasins 8 and 10. For the swimming exposure route, the cancer risk trend for OCPs is such that it is the highest in subbasin 10 followed by subbasin 8, subbasin 4, and subbasin 1, in decreasing order. Due to transboundary effects and long-distance transportation, it is possible that OCPs were transported from the upper course of the river to other sampling sites. Similar to the calculations of non-carcinogenic risk, upper bound cancer risk was also calculated using the mean concentration plus standard deviation (Figure S2 in the supplementary material).

## Discussion

Water is an essential part of all lives, for recreational, domestic, and agricultural uses. Oftentimes populations in rural and informal settlements in developing countries rely directly on the rivers for their everyday water uses. This water is generally used without prior treatment. Moreover, in these communities, people use water for recreational purposes. The uMsunduzi River in South Africa is one of the rivers with this kind of backdrop. Subbasins 1 and 10 are both in the rural parts of the uMsunduzi catchment, with subbasin10 in the headwaters and subbasin 1 by the confluence with the uMngeni River, ~15 km from the Inanda Dam. The Inanda Dam supplies Durban Metropolitan Municipality with a population estimated at ~ 3,228,000 with drinking water, and water quality concerns impact the cost of treatment.

### Key pollutants and their international significance

Overall, pesticides and nutrients have shown the highest non-carcinogenic risk in the studied catchment. Moreover, in all four subbasins (1, 4, 8, and 10), pesticides have shown cancer risk potential. Historically, in Africa, agriculture has been reported as the largest polluter, even more so than industries and municipalities (Olowu et al. 2010). Similar to the uMsunduzi catchment, the iSimangaliso Wetland Park and the uPhongolo floodplains, South Africa's largest floodplains and largest wetlands, have been found polluted with agricultural chemicals, further highlighting the threat to the country's biodiversity (Pieters and Horn 2020). As the country with the largest use of pesticides in sub-Saharan Africa, South Africa has likely misused many of the chemicals that are now classified as endocrine disrupting compounds (EDCs) (Horak et al. 2021); as evidenced by studies that have detected (anti-)oestrogenic and (anti-)androgenic activity in South African rivers, groundwater systems, and drinking water (Pieters and Horn 2020). In addition to being a concern to human health, impacts of EDCs have been discovered in wildlife species (Pieters and Horn 2020). Qu et al. (2015) discovered high potential of carcinogenic risk for humans exposed to OCPs in Ningde, Southeast China. In the study by Chen et al. (2020) in Shanghai, the ranking of the cancer risk caused by mistaken oral intake of OCPs was such that the risk was higher in adults than children. In the context of current study, the ranking is such that the risk in children is higher than in adults. The highest cancer risk was from drinking in subbasin 10 for children due to dieldrin.

Hazard quotients for PPCPs showed that the exposed population is unlikely to experience adverse health effects due to these chemicals, based on the concentrations used in this study. In the study by Kong et al. (2021), while the non-carcinogenic risk of antibiotics in drinking water was negligible, the ecological risks were high, based on the antibiotics concentrations in surface water around Lake Luoma in the north of Jiangsu province, China. The major concern with PPCPs is the endocrine disruption caused by natural and synthetic steroids and an increase in antibiotic resistance among microorganisms (Manickum and John 2014). Issues of antibiotic resistance, for instance, have been raised by many studies such as Cizmas et al. (2015); Adegoke et al. (2018), and Ben et al. (2019). Additionally, as an ecotoxicological risk, antibiotic residues in aquatic environments may pose threats to a variety of organisms at different trophic levels, with algae considered particularly sensitive to many antibiotics (Da Le et al. 2021). The chemicals continually added to the aquatic environment almost become "persistent" pollution (or pseudo-persistent), even if their half-lives are short, because their supply is continually replenished (Hernando et al. 2006; Patel et al. 2019), and PPCPs are an example of this. In the review by Adeleye et al. (2022), antibiotics and analgesics are the most frequently detected PPCPs in freshwater. This review by Adeleye et al. (2022) also shows that the analgesics represent the majority of the highest PPCP concentrations reported in Africa, including 107 μg/L of acetaminophen in the Ngong River, Kenya; 85 μg/L of ibuprofen in the uMsunduzi River, South Africa; and 62 μg/L of ibuprofen in the Umgeni River, South Africa. Analgesics (such as aspirin) are not recommended for children since they may affect liver functioning (Wexler 2014; The National Department of Health of South Africa 2020).

Madilonga et al. (2021) performed a risk assessment of heavy metals in the Mutangwi River in the Limpopo Province of South Africa. In that study, the non-carcinogenic risks were found to be lower than 1, for both children and adults, agreeing with a study done in Pakistan by Mohammadi et al. (2019) as well as the current study. Similarly, in Songnen Plain, Northeast China, Zhai et al. (2017) evaluated the non-carcinogenic risks associated with nutrients, explaining that the risk potential for children is higher than that for adults. There was a significant increase in phosphorus and nitrogen loadings at the Inanda Dam inflow between 2016 and 2020, and this is currently on an upward trend (Umgeni Water 2022). Due to nutrient enrichment, autotrophic growth occurs on a large scale, which has several consequences, such as biodiversity loss, oxygen depletion, algal toxin production, and taste/odour generation (Oberholster and Ashton 2008). Freshwater eutrophication is often caused by phosphate enrichment, and phosphate limitation is commonly used to control it (Isiuku and Enyoh 2020).

### Sources of uncertainty and their impact on study results

Even when the quantitative chemical risk assessment indicates that no adverse health effects are likely, it does not mean that the exposure to these chemicals is completely harmless. It is possible to develop new acute/chronic medical conditions or even exacerbate existing chronic conditions due to environmental exposure to pharmaceuticals. Moreover, the mixing of various chemical compounds may create a more toxic mixture than any one compound alone. De Souza et al. (2020) assert that a cocktail of pesticides exists in nature and could pose more toxic and adverse consequences to humans and animals exposed to them than a pesticide containing a single component. Such issues as ecotoxicity were not quantified in this study, and more work may be required on that subject. Riva et al. (2019) performed an environmental risk assessment of a mixture of emerging pollutants in surface water in a highly urbanised area in Italy using Risk Quotients. Risk Quotients consider the ratio of the expected exposure to the hazards of the mixture. Their findings indicated a potential cumulative risk for the substances that individually could be considered safe, highlighting the importance of taking the whole mixture of pollutants into account.

The chemical risk assessment in the current study was based on assumptions such as the exposure frequency and ingestion rates, which are likely to vary between individuals. Moreover, the concentrations considered in the risk assessment represent the water column and ignore the role of sediments from which the chemicals may re-enter the water column. The pollutant concentrations, with exception of the extensive long-term data for nutrients, are based on a very small number of samples that were gathered during focused campaigns, providing a snapshot in time. While for PPCPs, OCPs, and nutrients an upper bound risk estimate was calculated using the mean concentrations plus standard deviation, the same could not be performed for heavy metals.

### Exposure routes and vulnerabilities

Unequal access to water of reliable quality is both a cause and consequence of poverty in developing regions such as Africa (Olowu et al. 2010). Communities in rural subbasins (subbasins 10 and 1) of the uMsunduzi catchment are the most affected by pollution in the catchment due to their dependence on the river for domestic and recreational use. The highest HQ for children and adults exposed during drinking was found in Subbasin 1 due to exposure to phosphates. These communities are also exposed to a high risk of cancer due to pesticides.

As the resources for risk reduction are limited, it is necessary to prioritise risk-reduction measures by balancing risks, costs, and benefits. Techniques based on ecological engineering are preferable due to their high economic, environmental and ecological benefits, ease of maintenance, and because they are free from secondary pollution (Anawar and Chowdhury, 2020). Constructed wetlands, microbial dosing, ecological floating beds, and biofilm technologies are the most widely applicable ecological techniques (Anawar and Chowdhury, 2020). To control nutrient and chemical loads in catchments, chemical control technologies for agricultural runoff and household wastewater can be used (Shortle et al. 2020). Discussing the challenges and opportunities, including social, policy, institutional, and financial considerations, with all stakeholders will accelerate the adoption of reliable technologies to achieve system-level outcomes (Shortle et al. 2020).

This study makes a unique contribution by employing a quantitative chemical risk assessment methodology that not only considers a wide range of chemical contaminants and exposure pathways but also addresses the nuanced issue of water consumption variability between children and adults. This methodology has not been previously applied to this specific problem or within this study area, thus hindering the development of proper mitigation strategies. Consequently, this research has successfully provided valuable insights into the quantitative characterisation of chemical risks in surface waters.

## Conclusions

The population in the catchment of South Africa's uMsunduzi River is exposed to health risks through drinking untreated water from the river (in the rural areas), swimming (in the entire catchment), and canoeing (in the urban area). Organochlorinated pesticides were found to pose elevated cancer risks (except endrin), as well as cause long-term non-carcinogenic effects, in all subbasins. Heavy metals and pharmaceuticals and personal care products occurred at sub-risk levels. Phosphates could have ecological and health impacts, especially near the Inanda Dam. These findings aid catchment managers in prioritising high-risk areas when reducing chemical pollution in the uMsunduzi River.

### Supplementary Information

ESM 1 (DOCX 102 KB)

## Data Availability

The datasets used or analysed in the current study are available from the corresponding author upon reasonable request.

## References

[CR1] Adegoke A, Faleye A, Stenström TA (2018). Residual antibiotics, antibiotic resistant superbugs and antibiotic resistance genes in surface water catchments: Public health impact. Phys Chem Earth.

[CR2] Adeleye AS, Xue J, Zhao Y, Taylor AA, Zenobio JE, Sun Y, Han Z, Salawu OA, Zhu Y (2022). Abundance, fate, and effects of pharmaceuticals and personal care products in aquatic environments. J Hazard Mater.

[CR3] Adeyinka GC, Moodley B, Birungi G, Ndungu P (2019) Evaluation of organochlorinated pesticide (OCP) residues in soil, sediment and water from the Msunduzi River in South Africa. Environ Earth Sci 78(226). 10.1007/s12665-019-8227-y

[CR4] Agunbiade FO, Moodley B (2016). Occurrence and distribution pattern of acidic pharmaceuticals in surface water, wastewater, and sediment of the Msunduzi River, KwaZulu-Natal, South Africa. Environ Toxicol Chem.

[CR5] Anawar HM, Chowdhury R (2020) ‘Remediation of polluted riverwater by biological, chemical, ecological and engineering processes’. Sustainability (Switzerland) 12(17). 10.3390/su12177017

[CR6] ATSDR (2005). Toxicological Profile for Alpha-, Beta-, Gamma-, and Delta-Hexachlorocyclohexane.

[CR7] ATSDR (2007) Toxicological profile for heptachlor and heptachlor epoxide, Atlanta, Georgia 38091463

[CR8] ATSDR (2015). Toxicologic profile of hexachlorobenzene.

[CR9] ATSDR (2020). Toxicological Profile for Mirex and Chlordecone.

[CR10] Ben Y, Fu C, Hu M, Liu L, Wong MH, Zheng C (2019). Human health risk assessment of antibiotic resistance associated with antibiotic residues in the environment: A review. Environ Res.

[CR11] Chen C, Zou W, Chen S, Zhang K, Ma L (2020). Ecological and health risk assessment of organochlorine pesticides in an urbanized river network of Shanghai , China. Environ Sci Eur.

[CR12] Cizmas L, Sharma VK, Gray CM, McDonald TJ (2015). Pharmaceuticals and personal care products in waters: occurrence, toxicity, and risk. Environ Chem Lett.

[CR13] Da Le N, Hoang AQ, Hoang TTH, Nguyen TAH, Duong TT, Pham TMH, Nguyen TD, Hoang VC, Phung TXB, Le HT, Tran CS, Dang TH, Vu NT, Nguyen TN, Le TPQ (2021). Antibiotic and antiparasitic residues in surface water of urban rivers in the Red River Delta (Hanoi, Vietnam): concentrations, profiles, source estimation, and risk assessment. Environ Sci Pollut Res.

[CR14] de Souza RM, Seibert D, Quesada HB, de Jesus Bassetti F, Fagundes-Klen MR, Bergamasco R (2020). Occurrence, impacts and general aspects of pesticides in surface water: A review. Process Saf Environ Prot.

[CR15] Dorevitch S, Panthi S, Huang Y, Li H, Michalek AM, Pratap P, Wroblewski M, Liu L, Scheff PA, Li A (2011). Water ingestion during water recreation 5. Water Res.

[CR16] Dufour AP, Evans O, Behymer TD, Cantu R (2006) Water ingestion during swimming activities in a pool : A pilot study. J Water Health 4.4(2007). 10.2166/wh.2006.017 17176813

[CR17] DWAF (1996). South African Water Quality Guidelines: Volume 1 Domestic Use. Second Edi, Department of Water Affairs and Forestry.

[CR18] DWAF (1996). Water Quality Guidelines: Volume 2 Recreational Use. Second Edi, Department of Water Affairs and Forestry.

[CR19] Faleye AC, Adegoke AA, Ramluckan K, Fick J, Bux F, Stenström TA (2019). Concentration and reduction of antibiotic residues in selected wastewater treatment plants and receiving waterbodies in Durban South Africa. Sci Total Environ.

[CR20] Fryer M, Collins CD, Ferrier H, Colvile RN, Nieuwenhuijsen MJ (2006). Human exposure modelling for chemical risk assessment : a review of current approaches and research and policy implications. Environ Sci Policy.

[CR21] Genthe B, Kapwata T, Le Roux W, Chamier J, Wright CY (2018). The reach of human health risks associated with metals/metalloids in water and vegetables along a contaminated river catchment: South Africa and Mozambique. Chemosphere.

[CR22] Hernando MD, Mezcua M, Fernández-Alba AR, Barceló D (2006). Environmental risk assessment of pharmaceutical residues in wastewater effluents, surface waters and sediments. Talanta.

[CR23] Horak I, Horn S, Pieters R (2021) Agrochemicals in freshwater systems and their potential as endocrine disrupting chemicals: A South African context. Environ Pollut 268. 10.1016/j.envpol.2020.115718 10.1016/j.envpol.2020.115718 PMC7513804 33035912

[CR24] Isiuku BO, Enyoh CE (2020). Pollution and health risks assessment of nitrate and phosphate concentrations in water bodies in South Eastern, Nigeria. Environ Adv.

[CR25] Kong M, Bu Y, Zhang Q, Zhang S, Xing L (2021). Distribution , abundance , and risk assessment of selected antibiotics in a shallow freshwater body used for drinking water China. J Environ Manage.

[CR26] Lin K, Wang R, Han T, Tan L, Yang X, Wan M, Chen Y, Zhao T, Jiang S, Wang J (2023). Seasonal variation and ecological risk assessment of Pharmaceuticals and Personal Care Products (PPCPs) in a typical semi-enclosed bay — The Bohai Bay in northern China. Sci Total Environ.

[CR27] Madilonga RT, Edokpayi JN, Volenzo ET, Durowoju OS, Odiyo JO (2021) Water quality assessment and evaluation of human health risk in mutangwi river, Limpopo province, South Africa. Int J Environ Res Public Health 18(13). 10.3390/ijerph18136765 10.3390/ijerph18136765 PMC8296923 34202418

[CR28] Maleki A, Jari H (2021). Evaluation of drinking water quality and non-carcinogenic and carcinogenic risk assessment of heavy metals in rural areas of Kurdistan, Iran. Environ Technol Innov.

[CR29] Manickum T, John W (2014). Occurrence, fate and environmental risk assessment of endocrine disrupting compounds at the wastewater treatment works in Pietermaritzburg (South Africa). Sci Total Environ.

[CR30] Matongo S, Birungi G, Moodley B, Ndungu P (2015). Pharmaceutical residues in water and sediment of Msunduzi River, South Africa. Chemosphere.

[CR31] Mdletshe, M. (2019) ‘Msunduzi River spill: Small farmers worried they will not be compensated for “losses”’, GroundUp, 28 August, p. 6. Available at: https://www.groundup.org.za/article/msunduzi-river-spill-small-farmers-worried-they-will-not-be-compensated-losses/ Accessed 22 Aug 2022

[CR32] Mohammadi AA, Zarei A, Majidi S, Ghaderpoury A, Hashempour Y, Saghi MH, Alinejad A, Hosseingholizadeh N, Ghaderpoori M (2019). Carcinogenic and non-carcinogenic health risk assessment of heavy metals in drinking water of Khorramabad, Iran. MethodsX.

[CR33] Moloi M, Ogbeide O, Voua Otomo P (2020). Probabilistic health risk assessment of heavy metals at wastewater discharge points within the Vaal River Basin, South Africa. Int J Hyg Environ Health.

[CR34] Ngubane Z, Bergion V, Dzwairo B, Troell K, Amoah ID, Stenström TA, Sokolova E (2022) Water quality modelling and quantitative microbial risk assessment for uMsunduzi River in South Africa. J Water Health 20(4). 10.2166/wh.2022.266 10.2166/wh.2022.266 35482381

[CR35] Nyamukamba P, Moloto MJ, Tavengwa N, Ejidike IP (2019). Evaluating physicochemical parameters, heavy metals, and antibiotics in the Influents and final effluents of South African wastewater treatment plants. Pol J Environ Stud.

[CR36] Oberholster PJ, Ashton PJ (2008). An overview of the current status of water quality and eutrophication in South African Rivers and Reservoirs. Parliamentary Grant Deliverable.

[CR37] Olowu RA, Ayejuyo OO, Adewuyi GO, Adejoro IA, Akinbola TA, Osundiya MO, Onwordi CT (2010). Assessment of pollution trend of Oke Afa Canal Lagos, Nigeria. E-J Chem.

[CR38] Patel M, Kumar R, Kishor K, Mlsna T, Pittman CU, Mohan D (2019). Pharmaceuticals of emerging concern in aquatic systems: Chemistry, occurrence, effects, and removal methods. Chem Rev.

[CR39] Pieters R, Horn S (2020) Chemicals of concern in recreational waters: occurrence and assessment of potential human health risks in public swimming pools, Pretoria, South Africa

[CR40] Pironti C, Ricciardi M, Proto A, Bianco PM, Montano L, Motta O (2021). Endocrine-disrupting compounds: An overview on their occurrence in the aquatic environment and human exposure. Water.

[CR41] Qu C, Qi S, Yang D, Huang H, Zhang J, Chena W, Yohannes HK, Sandy EH, Yang J, Xing X (2015). Risk assessment and influence factors of organochlorine pesticides (OCPs) in agricultural soils of the hill region: A case study from Ningde, Southeast China. J Geochem Explor.

[CR42] Riva F, Zuccato E, Davoli E, Fattore E, Castiglioni S (2019). Risk assessment of a mixture of emerging contaminants in surface water in a highly urbanized area in Italy. J Hazard Mater.

[CR43] SANS (2015) SANS 241-1:2015 South African National Standards Drinking water Part 1 : Microbiological , physical , aesthetic, South Africa

[CR44] Sengar A, Vijayanandan A (2022). Human health and ecological risk assessment of 98 pharmaceuticals and personal care products (PPCPs) detected in Indian surface and wastewaters. Sci Total Environ.

[CR45] Shortle JS, Mihelcic JR, Zhang Q, Arabi M (2020). Nutrient control in water bodies: A systems approach. J Environ Qual.

[CR46] Shozi M (2015). Assessing the distribution of the sedimentary heavy metals in the Msunduzi River catchment, KwaZulu-Natal.

[CR47] Steyn M, Jagals P, Genthe B (2001). Assessment of microbial infection risks posed by ingestion of water during domestic water use and full-contact recreation in a mid-southern African region. Water Sci Technol.

[CR48] Taiwo AM (2019). A review of environmental and health effects of organochlorine pesticide residues in Africa. Chemosphere.

[CR49] The National Department of Health of South Africa (2020) Standard treatment guidelines and essential medicines list for South Africa, 7th edn. Pretoria, Republic of South Africa: The National Department of Health, Pretoria, Republic of South Africa Available at: https://www.knowledgehub.org.za/system/files/elibdownloads/2021-02/PrimaryHealthcareSTGs.and.EML.7th.edition-2020-v2.0.pdf Accessed 15 Jan 2022

[CR50] UNEP (2020). Stockholm Convention on persistent organic pollutants (POPS) - Texts and Annexes.

[CR51] USEPA (2009). USEPA Integrated Risk Information System (IRIS).

[CR52] Water U (2022) Infrastructure Master Plan 2022, Pietermaritzburg, South Africa Available at: https://www.umgeni.co.za/wp-content/uploads/2022/07/UWIMP_2022_Vol2.pdf. Accessed 26 Jun 2022

[CR53] Wexler P (2014). Encyclopedia of Toxicology. Third Edit, Encyclopedia of Toxicology.

[CR54] WHO (2008) WHO Guidelines for Drinking-Water Quality, 3rd edn, Geneva. 10.1248/jhs1956.35.307

[CR55] WHO (2010) WHO human health risk assessment toolkit: Chemical Hazards, Geneva, Switzerland

[CR56] WHO (2017). Guidelines for drinking-water quality: fourth edition incorporating the first addendum.

[CR57] WHO (2020). Human health risk assessment toolkit: chemical hazards. second edi.

[CR58] Wolmarans NJ, Bervoets L, Gerber R, Yohannes YB, Nakayama SM, Ikenaka Y, Ishizuka M, Meire P, Smit NJ, Wepener V (2021). Bioaccumulation of DDT and other organochlorine pesticides in amphibians from two conservation areas within malaria risk regions of South Africa. Chemosphere.

[CR59] Zamora-Ledezma C, Negrete-Bolagay D, Figueroa F, Zamora-Ledezma E, Ni M, Alexis F, Guerrero VH (2021). Heavy metal water pollution: A fresh look about hazards, novel and conventional remediation methods. Environ Technol Innov.

[CR60] Zhai Y, Zhao X, Teng Y, Li X, Zhang J, Wu J, Zuo R (2017). Groundwater nitrate pollution and human health risk assessment by using HHRA model in an agricultural area, NE China. Ecotoxicol Environ Saf.

