# Supplementary material for: Quantitative assessment of human health risks from chemical pollution in the uMsunduzi River, South Africa
Source: Environ Sci Pollut Res Int. 2023 Oct 24;30(55):118013–24. doi: 10.1007/s11356-023-30534-4 (PMC10682212; doi:10.1007/s11356-023-30534-4)
Supplement: Supplementary file 1 — (DOCX 102 KB) [file 11356_2023_30534_MOESM1_ESM.docx]

**Quantitative assessment of human health risks from chemical pollution in the uMsunduzi River, South Africa**

Zesizwe Ngubane^a^, Bloodless Dzwairo^a,b^, Brenda Moodley^c^, Thor Axel Stenström^b^, Ekaterina Sokolova^d,*^

^a^ Department of Civil Engineering, Midlands, Durban University of Technology, Pietermaritzburg, South Africa

^b^ Institute for Water and Wastewater Technology, Durban University of Technology, Durban, South Africa

^c^ School of Chemistry and Physics, University of KwaZulu-Natal, Westville Campus, Durban, South Africa

^d^ Department of Earth Sciences, Uppsala University, Uppsala, Sweden

*Corresponding author: ekaterina.sokolova@geo.uu.se

## Section 1

The tables presented in this section supplement the section on detected chemicals in the main article. These table show the chemicals’ effects on humans, status in South Africa, environmental persistence, cancer slope factor (where applicable), and reference doses.

*Table S 1 Organochlorinated pesticides detected in the uMsunduzi catchment; their human effects, status in South Africa (SA), environmental persistence, cancer slope factors (where applicable), and reference doses are presented below.*

| **Pesticide name** | **Human effects if the reference dose is exceeded.** | **Current Status**  **in South Africa** | **Environmental Persistence (Half-life in soil)** (Wexler, 2014) | **CSF**^[[1]](#footnote-1)^  **(1/mg/kg/day)**  (ATSDR, 2005, 2007, 2020, 2021b, 2022) | **Reference Dose (RfD)**^[[2]](#footnote-2)^  **(mg/kg/day)**  (ATSDR, 2005, 2007, 2020, 2021b, 2022) |
| --- | --- | --- | --- | --- | --- |
| aldrin (C_12_H_8_Cl_6_) | Myoclonic jerk, dizziness (Rani *et al.*, 2021). The target organs are the central nervous system and the liver (WHO, 2017). **Probable carcinogen** (ATSDR, 2021a) | Banned (1983) (DEA, 2011) | 2-15 years (changes to dieldrin) | 17 | 0.00003 |
| dieldrin (C_12_H_8_Cl_6_O) | Vomiting, disorder in muscle movement (Rani *et al.*, 2021).  **Probable human carcinogen** (ATSDR, 2021a) | Banned (1983) (DEA, 2011) | 5 years (Quinn et al., 2011) | 16 | 0.00005 |
| *o,p’-*DDE, *p,p’-*DDE | **Probable human carcinogen** (ATSDR, 2022) | Banned (1983) (DEA, 2011) | 2-30 years | 0.34 | 0.0003 |
| *o,p’*-DDD, *p,p’*-DDD | **Probable human carcinogen** (ATSDR, 2022) | Banned (1983) (DEA, 2011) | 2-30 years | 0.24 | 0.00003 |
| *o,p’*-DDT (C_14_H_9_Cl_5_) | Nausea, seizures (Rani *et al.*, 2021).  **Probable human carcinogen** (ATSDR, 2022) | Banned (1983) (DEA, 2011) | 2-30 years | 0.34 | 0.0005 |
| *p,p*-DDT (C_14_H_9_Cl_5_) | Nausea, seizures (Rani *et al.*, 2021), breast cancer (Wexler, 2014).  **Probable human carcinogen** (ATSDR, 2022) | Banned (1983) (DEA, 2011) | 2-30 years | 0.34 | 0.0005 |
| endrin (C_12_H_8_Cl_6_O) | Nausea, damage of CNS (Rani *et al.*, 2021) (WHO, 2017).  **Not carcinogenic** (ATSDR, 2021b) | Withdrawn in 1980 (DEA, 2011) | 12 years | - | 0.0003 |
| heptachlor (C_10_H_5_Cl_7_) | damage to the liver and central nervous system toxicity (WHO, 2017). P**robable human carcinogen** (ATSDR, 2007) | Banned (1983) (DEA, 2011) | 0.3-2.5 years (Quinn *et al.*, 2011) | 4.5 | 0.0005 |
| hexachlorobenzene  (HCB) (C_6_Cl_6_) | impairment of several organ systems including the kidneys, blood cells, and immune, endocrine, develop- mental, and nervous systems. In babies porphyria cutanea tarda, poor growth, arthritis, and enlarged thyroids. (Wexler, 2014). P**robable human carcinogen** (ATSDR, 2015) | Banned (1983) (DEA, 2011) | 3-6 years | 1.6 | 0.0008 |
| hexachlorocyclohexane (Lindane)  (HCH) (C_6_H_6_Cl_6_) | Liver and kidneys. **Probable human carcinogen** (ATSDR, 2005) | Banned (1983) (DEA, 2011) | 3-6 years | 6.3 | 0.0003 |
| mirex (C_10_Cl_12_) | Damage liver, CNS, and reproductive system (Rani et al., 2021)  **Probable human carcinogen** (ATSDR, 2020) | Was never registered in South Africa (DEA, 2011) | ~10 years | 0.93 | 0.0002 |

*Table S 2 Pharmaceuticals and personal care products detected in the uMsunduzi catchment; their human effects, and reference doses are presented below. (NR= Not recommended)*

| **Group** | **Pharmaceutical** | **RfD** (The National Department of Health of South Africa, 2020)  **Adults**  **(mg/kg/day)** | **RfD** (The National Department of Health of South Africa, 2020)  **Children (mg/kg/day)** | **Human effects if the RfD is exceeded** (Wexler, 2014) |
| --- | --- | --- | --- | --- |
| Antipyretics | Acetaminophen (C_8_H_9_NO_2_) | 4000 | NR | renal failure, liver failure, non-carcinogenic |
|  | Aspirin (C_9_H_8_O_4_) | 300 | NR | acute lung injury and pulmonary edema, acute renal failure, acute liver injury, and coagulopathies, non-carcinogenic |
|  | Diclofenac | 200 | 4 | Use of diclofenac tablets or capsules frequently or in high quantities, causes the risk of developing a stomach or intestinal ulcer. If there is a tiny chance of developing heart failure or kidney failure. |
|  | Ibuprofen | 2400 | 900 | gastritis, renal dysfunction, non-carcinogenic |
|  | Ketoprofen | 300 | NR | may result in stomach or intestinal ulcers, bleeding, or holes. |
| Stimulants | Caffeine (C_8_H_10_N_4_O_2_) | 2500 | NR | nausea, vomiting, hematemesis,  diarrhoea, and fever, metabolic acidosis, respiratory alkalosis, ketosis, hypokalaemia, and hyperglycaemia,  non-carcinogenic |
| Anti-epileptics | Carbamazepine (C_15_H_12_N_2_O) | 1600 | 15 | mouth sores, swollen lymph nodes, persistent vomiting, severe stomach/abdominal pain, yellowing eyes/skin, dark urine, change in the amount of urine, persistent or severe headache, fainting, fast/slow/irregular heartbeat, non-carcinogenic |
| Psychotics | Clozapine | 900 | NR | sedation, dizziness, insomnia, agitation, tardive dyskinesia, dysphoria,  dystonic reactions, tachycardia, syncope, anorexia, nausea, vomiting, constipation, diarrhoea, dyspepsia, non-carcinogenic |
| Antibiotics | Ampicillin | 2000 | 800 | severe stomach pain, diarrhoea; blisters, ulcers, or soreness in the mouth; skin rash, redness, or itching; fever, chills, sore throat, swollen glands, joint pain, or not feeling well; pale skin, cold hands and feet, non-carcinogenic |
|  | Ciprofloxacin (C_17_H_18_FN_3_O_3_) | 1500 | 1500 | neurotoxicity, altered mental status, hematologic effects, bleeding disorders, altered kidney function, crystalluria,  increases in liver enzymes, fatal hepatic failure, not carcinogenic |
|  | Erythromycin (C_37_H_67_NO_13_) | 4000 | 20 | gastric issues, profound cardiovascular toxicity, hepatic damage, not carcinogenic |
|  | Metronidazole (C_6_H_9_N_3_O_3_) | 1500 | 240 | headache, dry mouth,  nausea, and vomiting. dizziness,  vertigo, encephalopathy, seizures, and ataxia, not carcinogenic |
|  | Nalidixic acid | 1200 | 30 | increased intracranial pressure with bulging anterior fontanel, papilledema, and headache, not carcinogenic |
|  | Sulfamethoxazole | 480 | 80 | severe stomach pain, diarrhoea; skin rash; yellowing skin or eyes; a seizure; joint pain; increased or decreased urination, not carcinogenic |
|  | Sulfamethazine | 200 | NR | not carcinogenic |
|  | Trimethoprim | 480 | NR | diarrhoea, nausea, vomiting, stomach upset, loss of appetite, changes in taste, and headache,  not carcinogenic |
| Antihyperlipidemic | Bezafibrate | 600 | 160 | dizziness, muscle pain, tenderness, or weakness, not carcinogenic |

*Table S 3 Heavy metals and nutrients in the uMsunduzi catchment; their human effects, cancer slope factors (where applicable), and reference doses are presented below.*

| **Chemical** | **Human effects** (Wexler, 2014) | **CSF (1/mg/kg/day)**  (USEPA, 1988) | **RfD (mg/kg/day)**  (SANS, 2015) |
| --- | --- | --- | --- |
| Copper (Cu^2+^) | nonspecific toxic symptoms, a metallic taste, nausea, and  vomiting. Not classifiable as carcinogenic to human | n/a | 0.04 |
| Lead (Pb^2+^) | Anorexia, vomiting, malaise, and convulsions. Lead can cause neurological impairment in foetuses and young children (DWAF, 1996). Probable carcinogen | 0.0085 | 0.036 |
| Zinc (Zn^2+^) | Liver damage. Not carcinogenic. | n/a | 0.005 |
| Nitrate (NO_3_^-^) | relative risk of thyroid disorder and goitre rates in pregnant women, not carcinogenic | n/a | 0.36 |
| Phosphates  (PO_4_^3-^) | reduced lung function, aggravated asthmatic symptoms, and increased risk of emergency department visits, hospitalisations, and death in people who have chronic heart or lung diseases, not carcinogenic | n/a | 0.00002 |

## Section 2

The graphs presented in this section are supplementary to the results section. These graphs show chemical carcinogenic effects quantified using mean plus standard deviation of the concentrations.

Figure S 1 Hazard quotient for non-carcinogenic risk during swimming, drinking, and canoeing (top to bottom, respectively) calculated using mean plus standard deviation for both children (Ch) and adults (Ad).

Figure S 2 Cancer risk for carcinogenic risk during swimming, drinking, and canoeing (top to bottom, respectively) calculated using mean plus standard deviation for both children (Ch) and adults (Ad).

**References**

ATSDR (2005) *Toxicological Profile for Alpha-, Beta-, Gamma-, and Delta-Hexachlorocyclohexane*, *ATSDR’s Toxicological Profiles*. Atlanta, Georgia. doi: 10.1201/9781420061888_ch90.

ATSDR (2007) *Toxicological profile for heptachlor and heptachlor epoxide*. Atlanta, Georgia.

ATSDR (2020) *Toxicological Profile for Mirex and Chlordecone*, *ATSDR’s Toxicological Profiles*. Atlanta, Georgia. doi: 10.1201/9781420061888_ch117.

ATSDR (2021a) *Toxicological profile for aldrin/dieldrin draft version*. Atlanta, Georgia. Available at: www.regulations.gov.

ATSDR (2021b) *Toxicological Profile for Endrin*, *ATSDR’s Toxicological Profiles*. Atla. doi: 10.1201/9781420061888_ch81.

ATSDR (2022) *Toxicological Profile for DDT, DDE, and DDD*. Atlanta, Georgia. doi: 10.1201/9781420061888_ch13.

DEA (2011) *National implementation plan for the Stockholm Convention on Persistent Organic Pollutants*. South Africa.

Quinn, L. P., Vos, J. De, Roos, C., Bouwman, H., Kylin, H., Pieters, R. and Berg, J. Van Den (2011) *Pesticide use in South Africa: one of the largest importers of pesticides in Africa*, *Pesticides in the Modern World - Pesticides Use and Management*.

Rani, L., Thapa, K., Kanojia, N., Sharma, N., Singh, S., Grewal, A. S., Srivastav, A. L. and Kaushal, J. (2021) ‘An extensive review on the consequences of chemical pesticides on human health and environment’, *Journal of Cleaner Production*, 283, p. 124657. doi: 10.1016/j.jclepro.2020.124657.

SANS (2015) *SANS 241-1:2015 South African National Standards Drinking water Part 1 : Microbiological , physical , aesthetic*.

USEPA (1988) *Lead and compounds (inorganic ); CASRN 7439-92-1*, *Integrated Risk Information System*. Available at: https://cfpub.epa.gov/ncea/iris2/chemicallanding.cfm?substance_nmbr=277.

Wexler, P. (2014) *Encyclopedia of Toxicology*. Third Edit, *Encyclopedia of Toxicology*. Third Edit. London, UK: Elsevier.

1. CSF is the cancer slope factor, and it is defined as the risk generated by a lifetime average amount of one mg/kg/day of carcinogen chemical. [↑](#footnote-ref-1)
2. RfD is the reference dose, and it is defined as maximum acceptable oral dose of a toxic substance. [↑](#footnote-ref-2)
